# Gut-derived *Faecalibaculum rodentium* exerts anti-cancer effects on colorectal cancer by modulating PDPN-CLEC-2 signaling pathway

**DOI:** 10.1128/msystems.00148-25

**Published:** 2025-07-22

**Authors:** Xiaojuan Yang, Miao Liu, Ting Wang, Xinjuan Ma, Zhirong Guan, Bin Ma, Xiangguo Duan, Chunxia Su

**Affiliations:** 1 School of Inspection, Ningxia Medical University 105002, Yinchuan, China; 2 The First School of Clinical Medicine, Ningxia Medical University 105002, Yinchuan, China; 3 The Second School of Clinical Medicine, Ningxia Medical University 105002, Yinchuan, China; 4 School of Basic Medicine, Ningxia Medical University 105002, Yinchuan, China; 5 Department of Oncology Surgery, The First People’s Hospital of Yinchuan, Yinchuan, China; Argonne National Laboratory, Lemont, Illinois, USA

**Keywords:** acetate, colorectal cancer, CD8^+^T-cell immunity, CLEC-2, *Faecalibaculum rodentium*, PDPN

## Abstract

Dynamic changes in microbial composition are related to the progression of colorectal cancer (CRC); however, specific microbes with anti-CRC properties have not been identified. Here, we aimed to determine the effect of *Faecalibaculum rodentium* on CRC progression. In this study, the effect of *F. rodentium* was assessed in an azoxymethane (AOM)/dextran sulfate sodium salt (DSS)-induced CRC mouse model as well as in human CRC cell lines. Changes in anti-tumor immune responses were evaluated by flow cytometry. The functional metabolites produced by *F. rodentium* were identified by gas chromatography‒mass spectrometry (GC‒MS). The subcutaneously injected BALB/c mice with CT26 CRC cells were used to evaluate the effect of acetate produced by *F. rodentium*. We found that supplementation with *F. rodentium* suppressed CRC in an AOM/DSS-induced CRC mouse model as well as in the human CRC cell lines SW620 and HT-29. GC‒MS revealed that acetate is a functional tumor-suppressive metabolite produced by *F. rodentium*, and the tumor volume and tumor weight in the CRC model were inhibited by acetate administration. Mechanistically, *F. rodentium* produced acetate, which suppressed the expression of PDPN on CD8^+^ T cells and that of C-type lectin-like receptor 2 (CLEC-2) on tumor cells. As a consequence, CD8^+^ T-cell immunity was increased both *in vivo* and *in vitro*. In addition, we revealed that the acetate produced by *F. rodentium* can modulate podoplanin (PDPN)/CLEC-2/PI3K/AKT/mTOR signaling in CRC. In summary, *F. rodentium* produced acetate inhibits CRC by promoting CD8^+^ T-cell immunity and modulating the PDPN-CLEC-2 pathway. Our data indicate that *F. rodentium* is a potential probiotic for cancer prevention and treatment.

**IMPORTANCE:**

This study identified *Faecalibaculum rodentium* as a novel probiotic that suppresses colorectal cancer (CRC) via acetate, which enhances CD8^+^ T-cell immunity by blocking PDPN-CLEC-2 and induces tumor cell apoptosis through PI3K/AKT/mTOR pathway. These findings validated *F. rodentium* and acetate as dual-action therapeutic candidates through immune response activation and intrinsic tumor targeting by murine models and human CRC cell lines, thereby providing innovative strategies for CRC treatment.

## INTRODUCTION

Colorectal cancer (CRC) is the second leading cause of cancer death worldwide and the third most commonly diagnosed malignancy (1, 2), accounting for approximately 10% of all diagnosed cancer cases and cancer‐associated deaths per year (3, 4). Despite advancements in chemotherapeutic drugs and surgery, the long-term survival and cure rates of CRC patients are still poor (5). Therefore, identification of new therapeutic strategies is urgently needed to achieve effective treatment of CRC. Notably, accumulating evidence has revealed that polymorphic microbiomes, as hallmarks of cancer, have profound impacts on tumor progression and the response to anti-cancer therapies (6, 7).

The gut microbiota is a virtual metabolic and endocrine organ that comprises approximately 10^14^ microbial cells inside the gut (8). Dynamic changes in microbial composition have been shown to be related to CRC progression (9). Recent metagenomic studies have shown that *Fusobacterium nucleatum* (10), *Parvimonas micra* (11), *Helicobacter pylori* (12), *Bacteroides fragilis* (13), and *Peptostreptococcus anaerobius* (14) can promote the progression of CRC, while *Lactobacillus murinus*, *Bacteroides uniformis*, *Streptococcus thermophilus*, and *Streptococcus salivarius* can inhibit CRC progression (15). Recently, major efforts have been made to discover the anti-tumor functions of the gut microbiome. For example, Bell et al. (16) reported that *Lactobacillus reuteri* inhibits CRC by inducing oxidative stress and inhibiting protein translation. Winnie Fong et al. (17) reported that *Lactobacillus gallinarum*-derived metabolites increased the effectiveness of anti-PD1 therapy in patients with CRC by suppressing regulatory T cells. However, the potential beneficial bacteria involved remain largely unknown, and identification of specific microbes with anti-tumor properties may help to inhibit the progression of CRC.

*Faecalibaculum rodentium* is an anaerobic bacterium belonging to the Erysipelotrichaceae family. This species resides in the gut mucosa and helps to maintain gastrointestinal homeostasis (18). *F. rodentium* is lost during the early phases of tumorigenesis, and this change blocks tumor cell proliferation by reducing NFATc3 and calcineurin activation (19). Moreover, our previous study revealed that the abundance of *F. rodentium* decreased significantly in mouse with CRC (Fig. S1), which suggests that *F. rodentium* is a highly promising probiotic for CRC treatment. However, to date, whether *F. rodentium* can protect against CRC and the detailed mechanism underlying the therapeutic efficacy of *F. rodentium* have not been determined.

Therefore, the first aim of this study was to evaluate the therapeutic effects of *F. rodentium* in treating CRC in an azoxymethane (AOM)/dextran sulfate sodium salt (DSS)-induced CRC mouse model. The second aim of our study was to determine the molecular mechanism underlying the ability of *F. rodentium* to inhibit CRC by identifying crucial metabolites linked to CRC treatment.

Strong evidence supports the important role of the gut microbiota in reshaping host immunity, and different members of the microbiota have distinct abilities to induce T-cell activation and differentiation (20). Specifically, the gut microbiota-derived metabolites inosine and short-chain fatty acids (SCFAs), such as butyrate, acetate, and propionate, are absorbed across the intestinal epithelium and increase anti-tumor T-cell responses (21, 22). Notably, CD8^+^ T lymphocytes play a critical role in the tumor immune microenvironment (23). These cells can recognize tumor antigens and then target tumor cells after activation, and decreased infiltration or dysfunction of CD8^+^ T cells in the tumor microenvironment (TME) results in poor clinical outcomes in patients receiving many cancer therapies (24). Recently, extensive effort has been expended to identify the role of the microbiota in reshaping host CD8^+^ T responses (25). For example, *Megasphaera massiliensis*-derived pentanoate increases the production of effector cytokines in CD8^+^ T cells (26). These data led us to investigate whether *F. rodentium* promotes the CD8^+^ T-cell anti-tumor response and, therefore, inhibits the occurrence and development of CRC.

Notably, while CD8^+^ T cells are crucial for anti-tumor immunity, their function is often impaired by exhaustion within the TME, a state marked by sustained expression of immune checkpoint receptors such as PD-1, TIGIT, and CTLA-4. Consequently, targeting these receptors on CD8^+^ T cells represents a promising strategy to restore anti-tumor efficacy. Recent studies have identified podoplanin (PDPN), a lymphatic-specific transmembrane mucin, as a novel checkpoint ligand interacting with its receptor C-type lectin-like receptor 2 (CLEC-2). Although the PDPN/CLEC-2 axis suggests a unique immunoregulatory pathway, its role in *F. rodentium*-mediated CD8^+^ T-cell reprogramming and anti-tumor immunity is currently unknown.

Here, we showed that acetate produced by *F. rodentium* inhibits CRC. Specifically, we demonstrated that acetate produced by *F. rodentium* increases CD8^+^ T-cell immunity and modulates PDPN/CLEC-2/PI3K/AKT/mTOR signaling by downregulating PDPN expression on CD8^+^ T cells and CLEC-2 expression on tumor cells, resulting in the inhibition of CRC. Our data indicate that *F. rodentium* is a potential probiotic for cancer prevention and treatment.

## RESULTS

### Supplementation with *F. rodentium* suppresses the progression of CRC in mice

**Fig 1:**
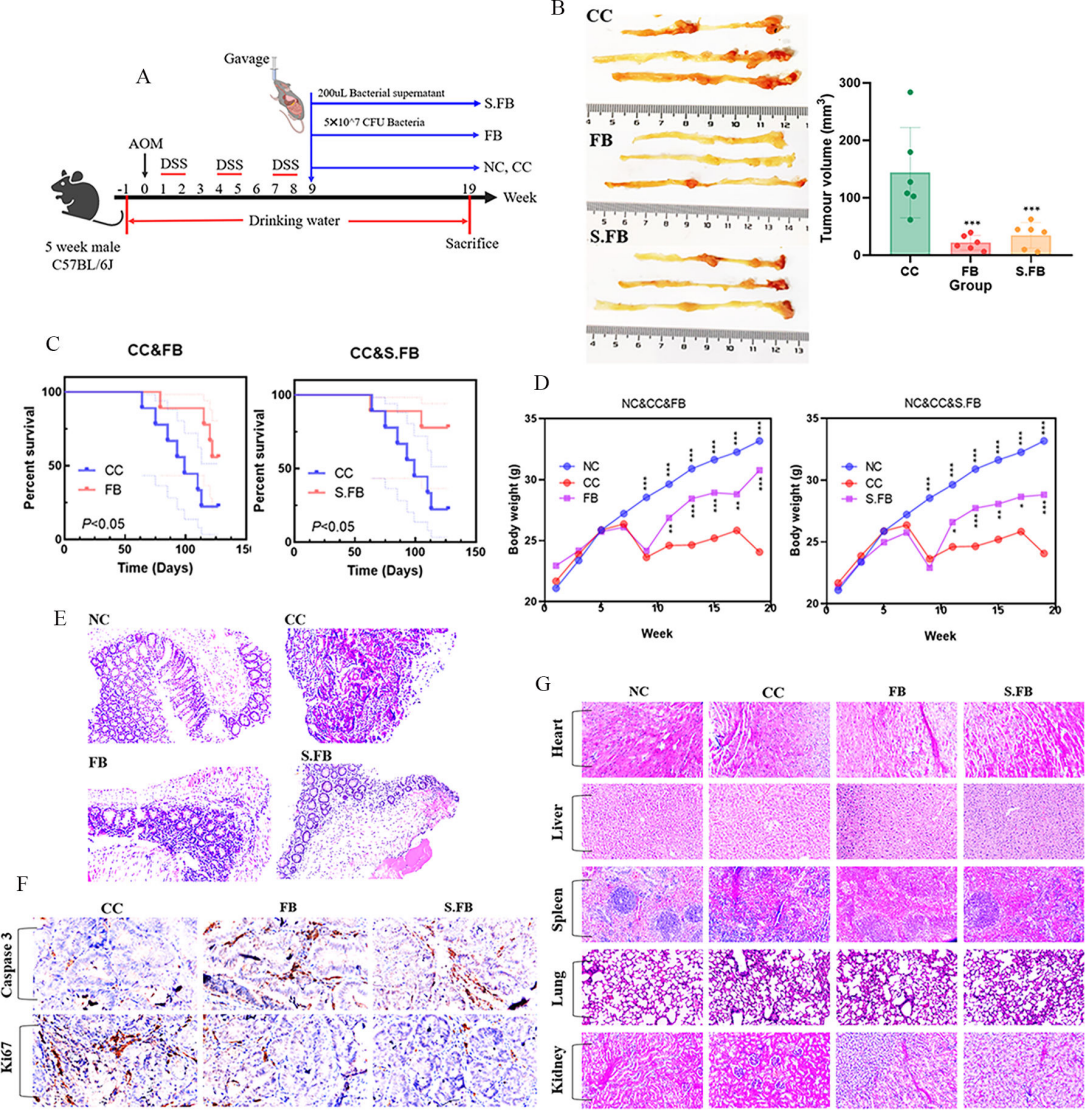
*F. rodentium*-secreted metabolites suppressed CRC in an AOM/DSS-induced CRC mouse model. (**A**) Schematic diagram showing the experimental design and timeline of the CRC mouse experiment, *n* = 9 mice per group. (**B**) Tumor volumes of the indicated groups (mm^3^). Tumor volumes were compared using one-way ANOVA. Data are presented as mean ± SD (*n* = 5 per group). (**C**) Survival of mice with CRC as detailed in panel **A**. Survival curves were analyzed by the Kaplan–Meier method, and statistical significance was determined using the log-rank test (*n* = 9 per group). A significant difference was observed between groups (*P* < 0.05). (**D**) Body weights of the indicated groups. Body weight curves were generated by pooling data from three independent experiments (*n* = 5 mice per group). At each time point, group differences were analyzed using one-way ANOVA. (**E**) Representative images of H&E staining of colorectal tissue. Scale bar: 50 µm. (**F**) Caspase-3 and Ki-67 immunohistochemical staining of tumor tissue. Scale bar: 50 µm. (**G**) Representative images of H&E-stained sections of the heart, liver, spleen, lung, and kidney. Scale bar: 50 µm. A two-tailed alpha level of 0.05 was used to determine statistical significance. *P*-values are presented as follows: ns, not significant; *P < 0.05; **P < 0.01; ***P < 0.001.

To determine whether *F. rodentium* can suppress CRC progression by producing metabolites, we established a mouse model of CRC induced by the AOM/DSS in this study. The CRC model mouse C57BL/6J was intragastrically administered the treatments, and the *Faecalibaculum rodentium* (FB) and supernatant of *F. rodentium* (S.FB) groups were treated with 5 × 10^7^ CFU of *F. rodentium* and 200 µL of the supernatant from *F. rodentium* three times a week for 10 consecutive weeks. The normal control (NC) and colorectal cancer control (CC) groups were treated with the same amount of phosphate-buffered saline (PBS) and used as the normal and CRC mouse controls, respectively (Fig. 1A).

The results showed that compared with PBS treatment, *F. rodentium* and its metabolites significantly reduced the tumor volume in the mice (Fig. 1B). Furthermore, the mice treated with *F. rodentium* and its metabolites had increased survival (Fig. 1C). Moreover, treatment with *F. rodentium* and its metabolites restored the body weight of the mice with CRC (Fig. 1D). Hematoxylin and eosin (H&E) staining revealed that the normal mouse colorectal tissue maintained intact tissue structures with well-aligned villi and abundant goblet cells. In contrast, colorectal tissues from carcinogen-induced mice (CC group) exhibited severe tissue disruption, including fragmented villi and a marked reduction in goblet cell populations. Notably, mice treated with *F. rodentium* and its metabolites demonstrated a notable increase in goblet cell density, restoration of tissue integrity, and attenuated inflammatory cell infiltration compared to the CC group (Fig. 1E). We further used immunohistochemistry (IHC) to determine the expression of Ki-67 and caspase-3 in mouse tumor tissues, and fewer Ki-67-positive cells and more caspase-3-positive cells were observed in the *F. rodentium* and metabolite-treated mice with CRC (Fig. 1F). To determine whether *F. rodentium* and its metabolites can damage other important organs in mice, we used H&E staining to analyze changes in the heart, liver, spleen, lung, and kidney tissues. We found that, compared with those in the normal mice, there were no obvious abnormalities in the heart, liver, spleen, lung, or kidney of the mice with CRC treated with *F. rodentium* and its metabolites. Thus, *F. rodentium* and its metabolites are safe for use in the treatment of CRC (Fig. 1G). Collectively, these results conclusively demonstrate that *F. rodentium* has a suppressive effect on CRC by producing anti-tumor metabolites.

### *F. rodentium* increases cytotoxic CD8^+^ T-cell function and downregulates the expression of PDPN on CD8^+^ T cells

As the microbiota has been shown to affect the progression of CRC via the modulation of host immune responses, we next tested whether the anti-tumor effect of *F. rodentium* and its metabolites is related to increased anti-tumor immunity. CD8^+^ T cells are the key executors of the anti-tumor immune response and act mainly via the classic perforin/granzyme release pathway and the release of TNF-α and IFN-γ to kill tumor cells and inhibit tumor development. To investigate whether *F. rodentium* and its metabolites promoted anti-tumor immunity in CD8^+^ T cells, we examined CD8^+^ T cells in the spleen, mesenteric lymph nodes (MLNs), and tumors.

**Fig 2:**
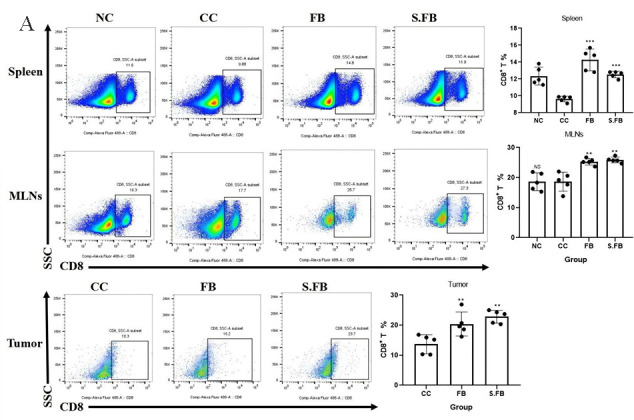
*F. rodentium* enhances the proportion of CD8^+^ T cells. (**A**) Representative flow cytometry data for CD8^+^ T-cell infiltration in the spleen, MLNs, and tumors. Plots were gated on T cells. The frequencies of CD8^+^ T cells were compared using one-way ANOVA. Data are presented as mean ± SD (*n* = 5 mice per group). A two-tailed alpha level of 0.05 was used to determine statistical significance. *P*-values are presented as follows: ns, not significant; *P < 0.05; **P < 0.01; ***P < 0.001.

Analysis of CD8^+^ T-cell dynamics showed that *F. rodentium* and its metabolites increased the proportion of CD8^+^ T cells in the spleen, MLNs, and tumors. These findings indicate that *F. rodentium* and its metabolites enhance anti-tumor immunity by directly boosting CD8^+^ T-cell activity in immune-suppressive environments, identifying them as potent immunomodulators (Fig. 2A). Additionally, we also analyzed Treg cells within the TME, and the results are presented in Fig. S2.

**Fig 3:**
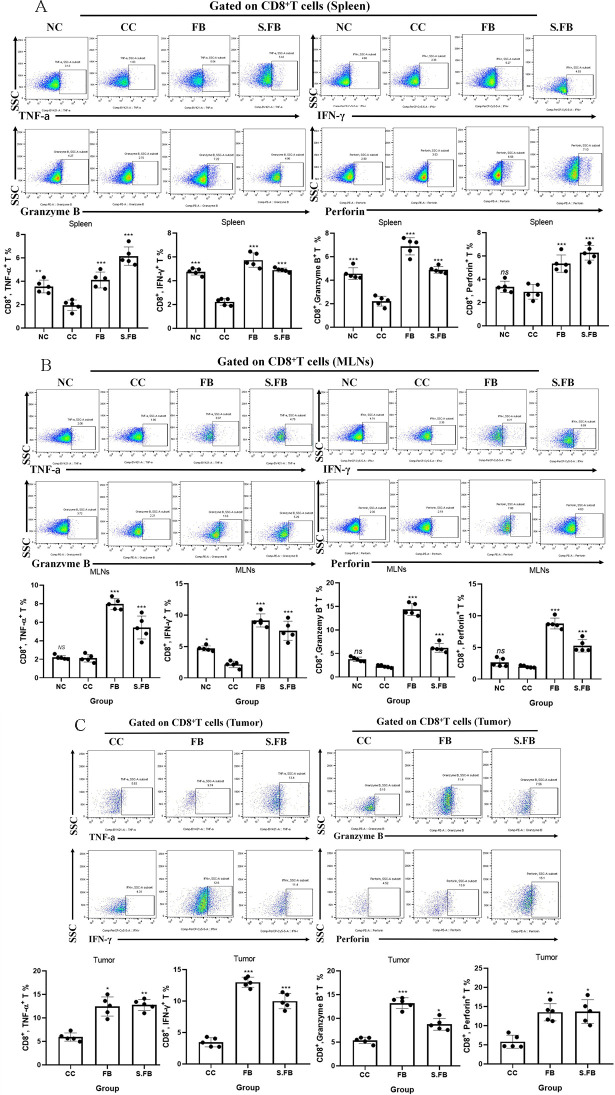
*F. rodentium* promotes CD8^+^ T cell-mediated anti-tumor immunity. (**A**) Representative flow cytometry data for CD8^+^ TNF-α^+^, CD8^+^ IFN-γ^+^, CD8^+^ granzyme-B^+^, and CD8^+^ perforin^+^ cell infiltration in the spleen. Plots were gated on CD8^+^ T cells. (**B**) Representative flow cytometry data for CD8^+^ TNF-α^+^, CD8^+^ IFN-γ^+^, CD8^+^ granzyme-B^+^ and CD8^+^ perforin^+^ cell infiltration in MLNs. Plots were gated on CD8^+^ T cells. (**C**) Representative flow cytometry data for CD8^+^ TNF-α^+^, CD8^+^ IFN-γ^+^, CD8^+^ granzyme-B^+^ cells and CD8^+^ perforin^+^ cells infiltrating in tumor tissues. Plots were gated on CD8^+^ T cells. The frequencies of CD8^+^ T-cell subsets in spleen, MLNs, and tumor tissues were compared using one-way ANOVA. Data are presented as mean ± SD (*n* = 5 mice per group). A two-tailed alpha level of 0.05 was used to determine statistical significance. *P*-values are presented as follows:ns, not significant; *P < 0.05; **P < 0.01; ***P < 0.001.

Further analysis revealed that, compared with those in the CRC control group, treatment with *F. rodentium* and its metabolites significantly promoted TNF-α, IFN-γ, granzyme-B, and perforin production in CD8^+^ T cells from the spleen, MLNs, and tumors (Fig. 3A through C), suggesting that *F. rodentium* and its metabolites may inhibit CRC by promoting CD8^+^ T-cell responses.

**Fig 4:**
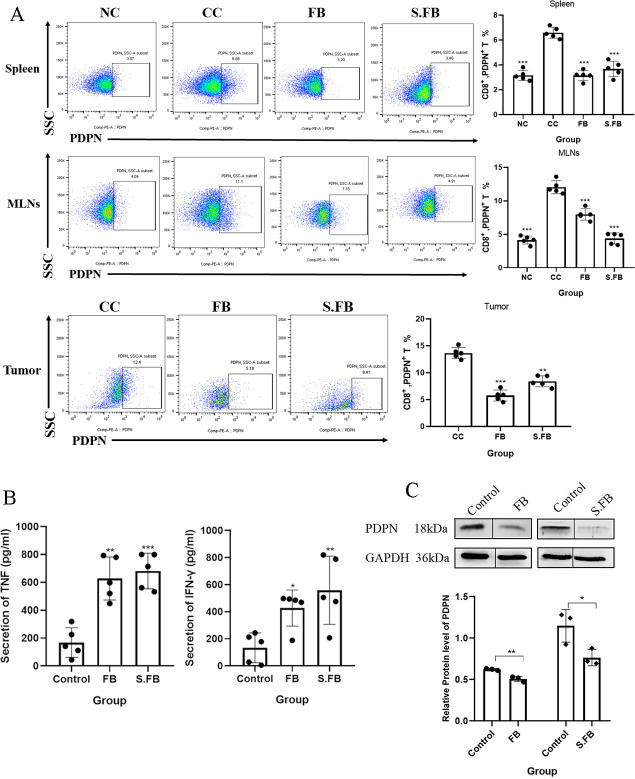
*F. rodentium* boosts CD8^+^ T-cell responses by decreasing PDPN levels. (**A**) Proportion of CD8^+^ PDPN^+^ T cells in the spleen, MLNs, and tumor tissues was determined by flow cytometry. Plots were gated on CD8^+^ T cells. The frequencies of CD8^+^ PDPN^+^ T cells in spleen, MLNs, and tumor tissues were compared using one-way ANOVA. Data are presented as mean ± SD (*n* = 5 mice per group). (**B**) Secretion of TNF-α and IFN-γ from Jurkat T cells was measured via ELISAs. Serum concentrations of TNF-α and IFN-γ were compared using one-way ANOVA. Data are presented as mean ± SD from five independent experiments. (**C**) Jurkat T cells were incubated with *F. rodentium* or its metabolites for 48 h. The protein levels of PDPN were tested via Western blotting. Representative immunoblots of PDPN and the loading control GAPDH are shown. Quantitative data are expressed as mean ± SD from three independent experiments. Statistical analysis was performed using an unpaired Student’s *t*-test. A two-tailed alpha level of 0.05 was used to determine statistical significance. *P*-values are presented as follows: ns, not significant; *P < 0.05; **P < 0.01; ***P < 0.001.

Additionally, immunohistochemical staining revealed that the expression of PDPN on lymphocytes in the tumor tissues of the mice with CRC induced by AOM/DSS was dramatically increased (Fig. S3A). PDPN is a heavily O-glycosylated small mucin-type transmembrane glycoprotein that can regulate the function of immune cells and is related to the malignant progression of several tumor types, but the association between PDPN and the microbiota is still unclear. To explore whether the improvement in CD8^+^ T-cell function is related to the regulation of PDPN expression by *F. rodentium* and its metabolites, we first analyzed the expression of PDPN on the surface of CD8^+^ T cells in the spleen, MLNs, and tumors by FCM. Notably, treatment with *F. rodentium* and its metabolites significantly inhibited the frequency of PDPN^+^ CD8^+^ T cells in the spleen, MLNs, and tumors compared to that in the CRC control group (Fig. 4A), indicating that the protein PDPN levels correlate negatively with the cytotoxic capacity of CD8^+^ T cells.

To further confirm the above results *in vitro*, we cocultured Jurkat T cells with *F. rodentium* and its metabolites. Consistent with these findings, enzyme-linked immunosorbent assay (ELISA) results showed that treatment with *F. rodentium* and its metabolites obviously increased TNF-α and IFN-γ production by Jurkat T cells (Fig. 4B). Moreover, Western blotting demonstrated that PDPN was downregulated in Jurkat T cells after treatment with *F. rodentium* and its metabolites (Fig. 4C). Taken together, these results demonstrated that the protein level of PDPN is related to the function of CD8^+^ T cells. *F. rodentium* produces metabolites that downregulate PDPN to promote the anti-tumor immunity of CD8^+^ T cells, which inhibits the progression of CRC.

### *F. rodentium* suppresses the proliferation, migration, and invasion of human CRC cells and promotes their apoptosis

**Fig 5:**
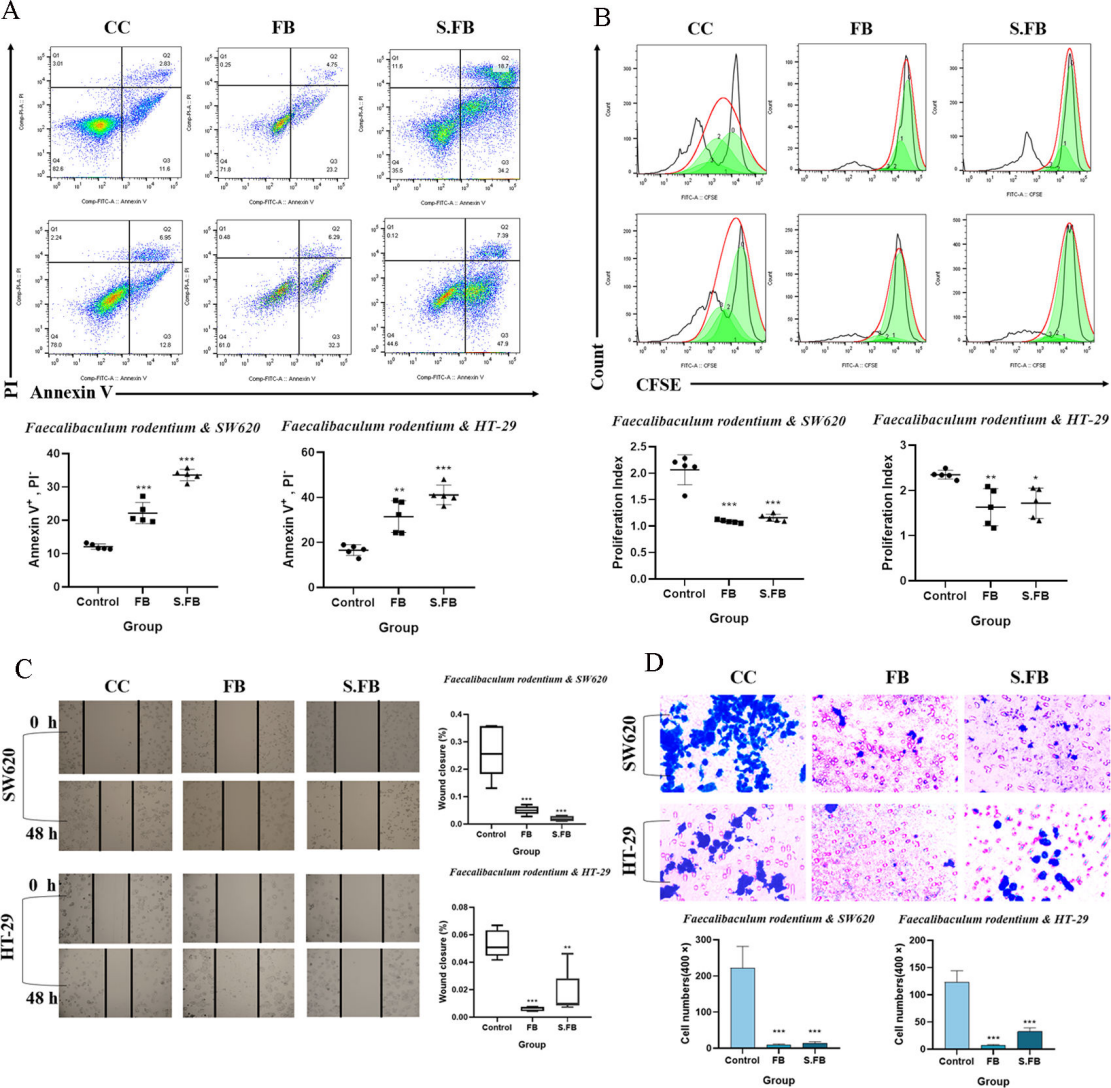
*F. rodentium* exhibits tumor-suppressive effects on CRC. (**A**) Treatment with *F. rodentium* and its metabolites induced cell apoptosis in SW620 and HT-29 cells. (**B**) Flow cytometry was used to examine the proliferation of SW620 and HT-29 cells that were treated with or without *F. rodentium* and its metabolites. (**C**) A scratch assay was performed to analyze the effects of treatment with *F. rodentium* and its metabolites on CRC cell migration. Scale bar: 200 µm. (**D**) Transwell assays showing the effects of treatment with *F. rodentium* and its metabolites on CRC cells for 48 h on cell invasion. Scale bar: 50 µm. Data are presented as mean ± SD of five independent experiments. Statistical significance was determined by one-way ANOVA. A two-tailed alpha level of 0.05 was used to determine statistical significance. *P*-values are presented as follows:ns, not significant; *P < 0.05; **P < 0.01; ***P < 0.001.

To further corroborate the potential anti-tumor effects of *F. rodentium* and its metabolites in CRC, we cocultured the human CRC cell lines SW620 and HT-29 with *F. rodentium* and its metabolites. *In vitro* cytotoxicity assays demonstrated that *F. rodentium* and its metabolites significantly suppress the viability of CRC cell lines SW620 and HT-29 in a concentration-dependent manner. In subsequent experiments, the doses of *F. rodentium* and its metabolites were selected based on their calculated IC_50_ values in CRC cells to ensure effective tumor suppression (Fig. S4). Annexin V/propidium iodide (PI) staining was performed to determine the effect of *F. rodentium* and its metabolites on apoptosis. Flow cytometry revealed that treatment with *F. rodentium* and its metabolites significantly increased the proportion of early apoptotic cells (annexin V^+^/PI^−^) in SW620 and HT-29 cells compared to the control group (Fig. 5A). Moreover, *F. rodentium* and its metabolites suppressed the proliferation of SW620 and HT-29 cells (Fig. 5B). Furthermore, compared with the control treatment, treatment with *F. rodentium* and its metabolites inhibited CRC cell migration and invasion (Fig. 5C and D). Taken together, these results suggested that *F. rodentium* can inhibit the growth of CRC cell lines by producing anti-tumor metabolites. Thus, our findings indicate that the tumor inhibitory effect of anti-tumor metabolites produced by *F. rodentium* in a CRC model is due to the dual effect of increased anti-tumor immune responses and direct tumor cell suppression.

### *F. rodentium* inhibits tumor growth by modulating the PDPN/CLEC-2/PI3K/AKT/mTOR pathway

**Fig 6:**
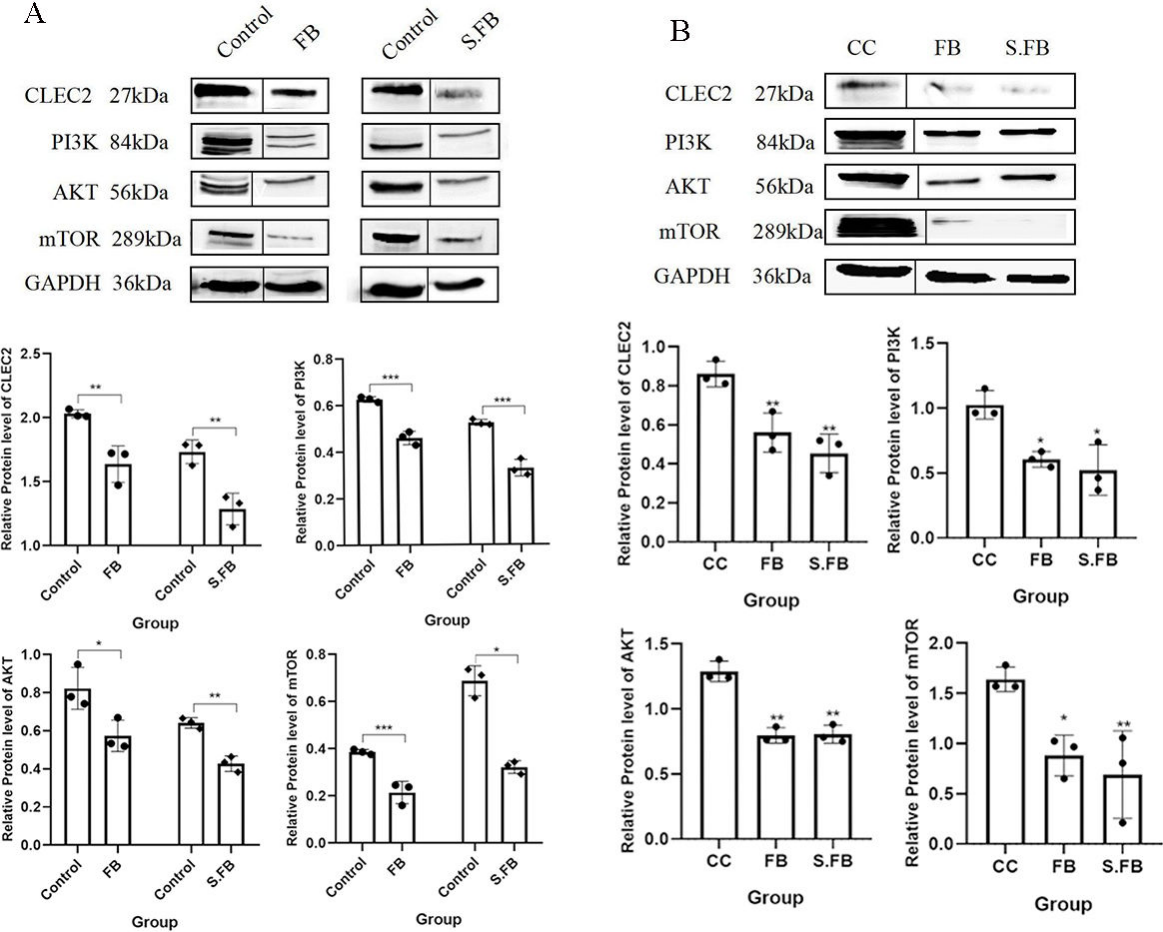
*F. rodentium* metabolites inhibit the PDPN/CLEC-2/PI3K/AKT/mTOR signaling pathway in CRC. (**A**) SW620 cells were incubated with *F. rodentium* or its metabolites for 48 h, after which the protein levels of CLEC-2, PI3K, AKT, and mTOR were measured via Western blotting. Representative immunoblots of the target protein and the loading control GAPDH are shown. Quantitative data are presented as mean ± SD from three independent experiments. Statistical analysis was performed using an unpaired Student’s *t*-test. (**B**) Protein levels and quantification of the CLEC-2/PI3K/AKT/mTOR pathway in tumor tissues from the AOM/DSS-induced CRC mouse model. Representative immunoblots of the target protein and loading control GAPDH are shown. Quantitative data are presented as mean ± SD from three independent experiments. Statistical significance was determined by one-way ANOVA. A two-tailed alpha level of 0.05 was used to determine statistical significance. *P*-values are presented as follows: ns, not significant; *P < 0.05; **P < 0.01; ***P < 0.001.

Previous studies have demonstrated that the binding of PDPN to its ligand CLEC-2 can contribute to cancer tumorigenesis, yet the function of the PDPN/CLEC-2 axis in CRC is unclear. In addition, we found that CLEC-2 was highly expressed in the tumor cells of mice with CRC induced by AOM/DSS (Fig. S3B and C), indicating that the level of CLEC2 in tumor cells may be negatively correlated with the progression of CRC. To identify the potential signaling pathway contributing to the suppressive effect of *F. rodentium* in CRC, we cocultured the human CRC cell line SW620 with *F. rodentium* and its metabolites for 48 h. As shown in Fig. 6A, CLEC-2 expression was downregulated in SW620 cells after treatment with *F. rodentium* and its metabolites. Further analysis revealed that the PI3K/AKT/mTOR signaling pathway was significantly inhibited after the administration of *F. rodentium* and its metabolites (Fig. 6A). We then validated this pathway in an AOM/DSS-induced CRC mouse model. Consistent with the *in vitro* results, treatment with *F. rodentium* and its metabolites significantly suppressed CLEC-2 protein expression and the PI3K/AKT/mTOR signaling pathway in tumor tissues (Fig. 6B). Taken together, these findings indicate that the protective effect of *F. rodentium* is mediated at least in part through the inhibition of PDPN/CLEC-2/PI3K/AKT/mTOR signaling.

### The SCFA acetate is the functional tumor-suppressive metabolite produced by *F. rodentium*

**Fig 7:**
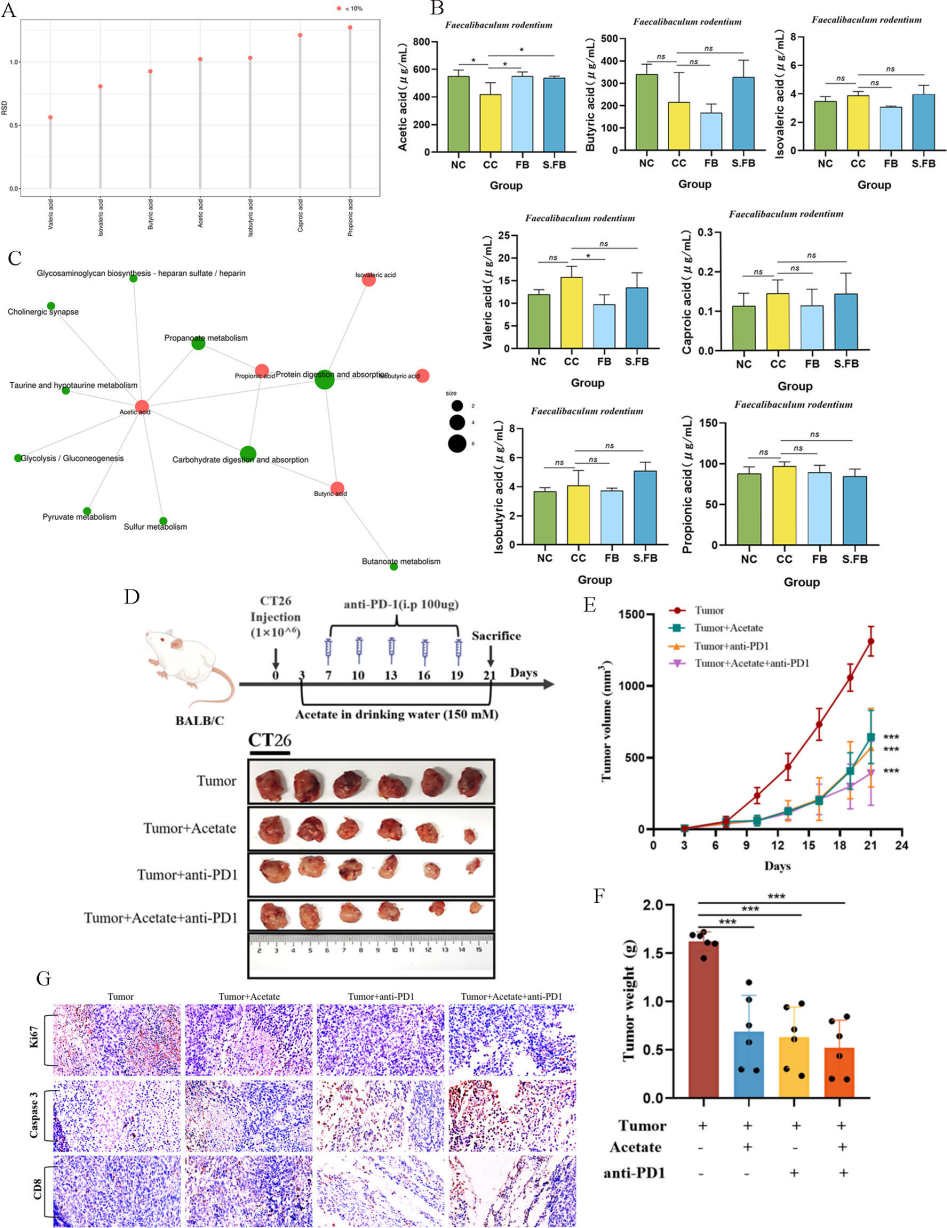
Measurement of the contents of SCFAs in colonic contents. SCFAs in the colonic contents were determined by GC‒MS. (**A**) Analysis of QC data; RSD <15%. (**B**) Concentrations of acetic acid, butyric acid, isovaleric acid, valeric acid, caproic acid, isobutyric acid, and propanoic acid in samples from the colonic contents of mice. Concentrations of SCFAs were compared using one-way ANOVA. Data are presented as mean ± SD (*n* = 3 per group). (**C**) Kyoto Encyclopedia of Genes and Genomes (KEGG) analysis. (**D**) Schematic diagram showing the experimental design and timeline of subcutaneous CRC murine models. (**E**) Tumor volume in the CRC model. Tumor volumes were compared using one-way ANOVA. Data are presented as mean ± SD (*n* = 6 per group). (**F**) Tumor weight in the CRC model.Tumor weights were compared using one-way ANOVA. Data are presented as mean ± SD (*n* = 6 per group). (**G**) Caspase-3, Ki-67, and CD8 immunohistochemical staining of tumor tissue. Scale bar: 50 µm. A two-tailed alpha level of 0.05 was used to determine statistical significance. *P*-values are presented as follows: ns, not significant; *P < 0.05; **P < 0.01; ***P < 0.001.

Because the metabolites produced by *F. rodentium* suppressed CRC, we identified the specific component of the gut microbial metabolites that was the functional metabolite produced by *F. rodentium*. Previous studies have demonstrated that microbiome-derived SCFAs affect the progression of CRC, and SCFA levels were reported to decrease in CRC. To verify whether *F. rodentium* produces SCFAs in CRC, we performed targeted SCFA metabolomic analysis of the colonic contents of the mice with AOM/DSS-induced CRC with or without treatment with *F. rodentium* and its metabolites. Quality control (QC) samples were subjected to QC analysis during GC‒MS detection, and the relative standard deviation (RSD) values of the targeted metabolites were all less than 15%, indicating that the experimental data were credible in this study (Fig. 7A). Next, we determined the concentrations of SCFAs, including acetic acid, butyric acid, isovaleric acid, valeric acid, caproic acid, isobutyric acid, and propanoic acid, in the different groups. Compared with the control treatment, treatment with *F. rodentium* and its metabolites significantly increased the level of acetic acid (Fig. 7B). Moreover, the increase in acetic acid was involved in many metabolic pathways that are critical for host physiology, including protein digestion and absorption, pyruvate metabolism, and glycolysis/gluconeogenesis (Fig. 7C).

To further ascertain the role of acetate in mice with CRC, we subcutaneously injected BALB/c mice with CT26 CRC cells and exposed them to sodium acetate through the drinking water to evaluate the effect of acetate in anti-cancer therapy. Another group of mice received acetate in the drinking water, followed by anti-PD‐1 therapy (Fig. 7D). The data suggest that, compared with those of the control mice, the tumor volume and tumor weight in the CRC model were inhibited by acetate administration (Fig. 7E and F). In addition, fewer Ki-67-positive cells and more caspase-3- and CD8-positive cells were observed in the acetate-treated mice with CRC than in the control mice (Fig. 7G). Consistent with these findings, in the CRC model, the addition of anti-PD‐1 therapy and acetate in combination produced greater anti-tumor effects than the individual agents alone. Taken together, these data suggest that acetate is a functional tumor-suppressive metabolite produced by *F. rodentium* and that the combination of acetate with immune checkpoint inhibitors has anti-tumor effects.

## DISCUSSION

First, we showed that *F. rodentium* suppresses the progression of CRC by producing anti-tumor metabolites. We found that *F. rodentium*-produced metabolites significantly promoted TNF-α, IFN-γ, granzyme-B, and perforin production in CD8^+^ T cells from the spleen, MLNs, and tumors by suppressing the expression of PDPN on CD8^+^ T cells, suggesting that CD8^+^ T cells are the key immune cells regulated by *F. rodentium*. Furthermore,through *in vitro* and *in vivo* experiments, we demonstrated that anti-tumor metabolites produced by *F. rodentium* can also modulate PDPN/CLEC-2/PI3K/AKT/mTOR signaling in CRC. Finally, we revealed that acetate is the functional tumor-suppressive metabolite produced by *F. rodentium*. Overall, *F. rodentium* inhibits the progression of CRC by promoting CD8^+^ T-cell immunity and modulating the PDPN-CLEC2 pathway. To our knowledge, this study is the first to identify the effects and mechanisms of *F. rodentium* in CRC.

In our study, *F. rodentium* produced acetate, which not only increased CD8^+^ T-cell immunity but also inhibited CRC cell growth by suppressing CLEC2 expression on CRC cells. The exploration of the beneficial effects of bacterial metabolites has focused mainly on one type of cell, especially immune cells. Consistent with our findings, acetate acts as a metabolic immunomodulator by bolstering CD8^+^ T-cell effector function and potentiating anti-tumor immunity in breast cancer (27). In addition, consistent with our results, *Bifidobacterium pseudolongum*-generated acetate reached the liver via the portal vein, where it suppressed the IL-6/JAK1/STAT3 signaling pathway in hepatocytes, thereby preventing hepatocellular carcinoma (28). Notably, the two studies mentioned above suggest that bacterial metabolites can not only inhibit tumors by boosting the function of CD8^+^ T lymphocytes but also directly influence tumor cells, which is consistent with our findings. Importantly, the novelty of our study is that the tumor inhibitory effect of anti-tumor metabolites produced by *F. rodentium* in a CRC model is due to the dual effect of an increased anti-tumor immune response and direct tumor cell suppression. Further research should be conducted in a number of well-known CRC models in rodents.

Notably, we identified two transmembrane receptors, PDPN and CLEC2, that are related to the anti-tumor effect of *F. rodentium*. PDPN is a type I transmembrane glycoprotein expressed in kidney podocytes, skeletal muscles, lungs, hearts, myofibroblasts, osteoblasts, mesothelial cells, and lymphatic endothelial cells (29, 30). The upregulation of PDPN has been observed in a variety of human cancers, including brain cancer, breast cancer, lung cancer, and mesothelioma, and is associated with poor prognosis (31, 32). CLEC-2 is a platelet-activating type II transmembrane receptor and is highly expressed on megakaryocytes and platelets (33). Numerous studies have indicated that PDPN and CLEC-2 have various physiological and pathological roles and that the PDPN-CLEC2 pathway is crucial for carcinogenesis, metastasis, and prognosis (32, 34). Interestingly, in the present study, we observed that PDPN on lymphocytes and CLEC-2 on tumor cells were dramatically increased in CRC, which was possibly related to the progression of CRC. Furthermore, treatment with *F. rodentium* specifically decreased the expression of CLEC2 on tumor cells and PDPN on CD8^+^ T cells. Thus, we demonstrated that *F. rodentium* decreases PDPN-CLEC-2 signaling, which further contributes to CRC inhibition. This is a promising result, and targeting PDPN or CLEC-2 could lead to novel possibilities for cancer treatment and prevention.

Additionally, our study provided extensive evidence for the tumor inhibitory effect of *F. rodentium* in an AOM/DSS-induced CRC mouse model. Recently, due to the rapid development of next-generation sequencing technology, accumulating evidence has clarified the complex role of the microbiota in cancer development and treatment response (15, 35). In recent years, an increasing number of studies have focused on how to regulate cancer through the use of specific microorganisms, and microbial-based cancer treatment has made substantial progress (36, 37). For example, *Lactobacillus rhamnosus* GG increased the numbers of tumor-infiltrating dendritic cells (DCs) and T lymphocytes, which improved the anti-tumor effectiveness of anti-programmed cell death 1 (PD-1) immunotherapy (38). *S. thermophilus* secretes galactosidase to induce oxidative phosphorylation and downregulate Hippo pathway kinases, which largely mediate the anti-cancer effects (39) of *S. thermophilus*. Consistent with previous studies on the importance of particular bacteria in cancer prevention and treatment, our findings identified a novel probiotic, *F. rodentium*, which has the potential to suppress CRC. However, this approach does not cause damage to other organs or tissues, indicating that it is safe for use in CRC treatment.

Here, we demonstrated that *F. rodentium* mediated anti-CRC effects via acetate secretion and that acetate potentiated anti-PD-1 therapy *in vivo*. The underlying mechanism relied on a synergistic strategy to enhance anti-tumor immunity through the convergence of two distinct effects. First, acetate, acting as a SCFA, activated GPR43, driving metabolic reprogramming in CD8^+^ T cells and directly boosting their effector function. Second, acetate reduced PDPN expression, disinhibiting CLEC2 signaling and complementing anti-PD-1’s blockade of the PD-1/PD-L1 axis. This combined checkpoint inhibition reduced CD8^+^ T-cell exhaustion, promoted clonal expansion, and more broadly reversed immunosuppression. Consequently, this synergistic action significantly enhanced CD8^+^ T cell-mediated anti-tumor immunity, thereby improving the therapeutic outcome of anti-PD-1 therapy.

Although murine and cell line data are promising, clinical translation faces challenges. First, the complexity of the human gut microbiome and the dynamic balance of host-microbe interactions may significantly impact the colonization efficiency of exogenous *F. rodentium* and the local concentration of acetate. Individual variations in the gut environment may further limit the universality of this treatment. Second, acetate is readily metabolized or absorbed *in vivo*, which may limit its effective concentration in the TME. Targeted delivery systems, such as nanocarriers or engineered probiotics, are needed to optimize its bioavailability. Importantly, the long-term safety of exogenous probiotics, including the risk of gut dysbiosis or bacteremia, and the potential off-target effects of metabolic interventions require rigorous evaluation through systematic toxicology studies and early-phase clinical trials. This study proposes a novel strategy for CRC by targeting the microbiome-immune-metabolic axis. However, clinical translation necessitates the integration of multi-omics and precision medicine to address species-specific challenges and delivery hurdles, ultimately translating mechanistic findings into clinical benefit.

A limitation of this study is that, while we discovered that *F. rodentium* has dual effects via an increased anti-tumor immune response and direct tumor cell suppression in CRC, it is unclear whether *F. rodentium* has a greater effect on CD8^+^ T cells or tumor cells. Furthermore, the majority of our studies were conducted in mice, and it remains to be determined whether *F. rodentium* affects anti-tumor CD8^+^ T-cell immunity in humans. Finally, it is unclear whether different doses of *F. rodentium* could affect different immune and tumor cells in the TME. In the future, methods to determine the dose of *F. rodentium* supplemented to mice should be established.

Taken together, our study demonstrated that *F. rodentium* produced acetate, which suppressed the expression of PDPN on CD8^+^ T cells, and that of CLEC2 on tumor cells. As a consequence, CD8^+^ T-cell immunity was enhanced both *in vivo* and *in vitro*. In addition, we revealed that the acetate produced by *F. rodentium* can modulate PDPN/CLEC-2/PI3K/AKT/mTOR signaling in CRC. In summary, *F. rodentium* inhibits the progression of CRC by promoting CD8^+^ T-cell immunity and modulating the PDPN-CLEC2 pathway. Our findings might be helpful in determining potential treatment strategies and targets for CRC patients.

## MATERIALS AND METHODS

### Animal experiments

Male C57BL/6J mice were utilized for this study and purchased from the Ningxia Medical University Laboratory Animal Center. All mice were between 5 and 6 weeks of age at the time of use. The mice were housed at an ambient temperature of approximately 22°C under a 12 h light-dark cycle at Ningxia Medical University. Mice were given a cycle of one single intraperitoneal injection of AOM (Cat. #A5486; Sigma‒Aldrich [Shanghai] Trading Co., Ltd., 10 mg/kg body weight) during the first week. Next, each group of mice was given 2% DSS (Cat. #42867; Sigma‒Aldrich [Shanghai] Trading Co., Ltd.) and then allowed to drink normal water for 14 days; this process was repeated for three cycles. The mice in the FB and S.FB groups were treated with 5 × 10^^7^ CFU of *F. rodentium* and 200 µL of the supernatant from *F. rodentium* three times per week for 10 consecutive weeks. The mice in the NC and CC groups were treated with the same volume of PBS and defined as the normal and CRC mouse controls, respectively.

For establishment of subcutaneous CRC murine models, 4- to 5-week-old female BALB/c mice were purchased from Ningxia Medical University Laboratory Animal Center and were subcutaneously injected with 1 × 10^6^ CT-26 CRC cells. Once the tumors formed, the mice were randomly allocated to different experimental groups. For mice treated with acetate, sodium acetate (Sigma, 150 mM) was added to the drinking water, and the water was changed every 2 days throughout the experiment. For mice treated with anti-PD-1, mice were intraperitoneally injected with 100 µg of anti-PD-1 (Bio X Cell, BP0033-2) every 3 days (100 µg per mouse, total of five injections).

### Bacterial strain and culture

*F. rodentium* was purchased from Ningbo Mingzhou Biotechnology Co., Ltd. (JCM 30274) and cultured in modified PYG medium (modified, Cat. #MD090B, ShanDong Tuopu Bioengineering Co., Ltd., China) under anaerobic conditions at 37°C. When *F. rodentium* reached a specific optical density, the bacterial culture supernatant was centrifuged at 5,000 rpm for 10 minutes and filtered through a 0.2 µm pore-size filter to obtain the metabolites of *F. rodentium* (S.FB). The concentration of bacteria was measured by a turbidimeter (MB-5271, Changchun LePu Technology Co., Ltd., China).

### Cell culture

Human CRC cell lines SW620 (ATCC; CCL-227) and HT-29 (ATCC; HTB-38), along with the murine CRC cell line CT-26 (ATCC; CRL-2638), were obtained from the American Type Culture Collection (ATCC, Manassas, VA, USA). All the cell lines were grown in Dulbecco’s modified Eagle’s medium (VivaCell) supplemented with 10% fetal bovine serum (FBS, VivaCell) and 1% penicillin/streptomycin. Jurkat T cells (ATCC; TIB-152) were obtained from the ATCC and grown in RPMI 1640 (VivaCell) supplemented with 1% penicillin‒streptomycin and 10% FBS. All the cells were cultured at 37°C in a humidified atmosphere containing 5% CO^2^.

### Cell apoptosis

SW620 and HT29 cells were exposed to *F. rodentium* and its metabolites for 48 h and were harvested. An FITC Annexin V Apoptosis Detection Kit I (Cat. #556547, BD Pharmingen) was used to assess the cells after incubation with FITC annexin V and PI for 15 minutes at room temperature. Then, the cells were analyzed via a flow cytometer.

### Cell proliferation

Cell proliferation was assessed with CellTrace Cell Proliferation Kits (Cat. #C34570, Invitrogen). SW620 and HT29 cells were harvested and incubated with carboxyfluorescein diacetate succinimidyl ester (CFSE) for 15 minutes at 37°C, after which 5% FBS was added to stop the staining for 10 minutes. The stained cells were exposed to *F. rodentium* and its metabolites for 48 h and then analyzed by a flow cytometer.

### Cell migration assay

A scratch assay was used to analyze cell migration *in vitro*. When the SW620 and HT29 cells were more than 90% confluent, a wound was created on the surface of the plates using a 10 µL pipette tip, after which the cells were photographed under a microscope. The cells were incubated with *F. rodentium* and its metabolites for 48 h and then imaged with a microscope.

### Cell invasion assay

For the invasion assay, 8.0 µm PC membrane Transwells (BIOFIL, Guangzhou JetBio-Filtration Co., Ltd., China) were added to a 24-well plate to produce upper and lower chambers, and Matrigel was used for cell invasion assays (Cat. #0827065 Shanghai Nova Pharmaceutical Technology Co., Ltd., China). The lower chamber of the 24-well plate was filled with 750 µL of medium containing 10% FBS, and the Transwell chamber was filled with *F. rodentium* and its metabolites and incubated with HT-29 and SW620 cells for 48 h. After fixation, staining, and counting under a microscope, the invasive ability of the cells was determined.

### Enzyme-linked immunosorbent assay

Jurkat T cells were treated with *F. rodentium* and its metabolites for 48 h. The levels of the cytokines TNF (BD Pharmingen) and IFN-γ (Invitrogen) in the supernatants were measured via ELISAs according to the manufacturer’s instructions.

### Histopathological analysis

Heart, liver, spleen, lung, kidney, and colorectal tissues were fixed in 4% paraformaldehyde for 48 h at 4°C and then embedded in paraffin. Next, 3.5 µm sections were stained with H&E for pathological analysis.

For immunohistochemistry, tumor tissues were fixed in 4% paraformaldehyde for 48 h at 4°C before being embedded in paraffin. Paraffin-embedded tissue sections were incubated with anti-caspase-3 (Thermo Fisher, Catalog #700182) or anti-Ki-67 (Thermo Fisher, Catalog #MA5-14520) overnight at 4°C. The following day, the sections were washed and incubated at 37°C for 30 minutes with secondary antibodies labeled with horseradish peroxidase. Immunostaining was detected with diaminobenzidine, and the sections were counterstained with haematoxylin, as directed by the manufacturer.

### Western blot

Cell samples and tumor tissues were lysed in an ice bath for 30 minutes with Whole Cell Lysis Assay (KeyGEN BioTECH, Jiang Su, China). The supernatants were collected, and a BCA Protein Assay Kit (KeyGEN BioTECH, Jiang Su, China) was used to measure the protein concentrations. An SDS‒PAGE gel preparation kit (KeyGEN BioTECH, Jiang Su, China) was used to separate the denatured proteins, which were subsequently transferred to Immobilon-P Transfer Membranes. The polyvinylidene fluoride (PVDF) membranes were blocked in 5% skim milk (Catalog #232100, BD) in Tris-buffered saline with Tween-20 (TBST) (Catalog #SW111-02, SEVEN, SEVEV Innovation [Beijing] Biotechnology Co., Ltd.) for 1.5 h at room temperature, incubated with primary antibodies overnight at 4°C and then treated with horseradish peroxidase-conjugated secondary antibodies (Proteintech, Wuhan Sanying Biotechnology Co., Ltd., China). After phosphate-buffered saline with Tween-20 (PBST) washes, the proteins were visualized using an enhanced chemiluminescence Western blotting detection kit (Advansta, USA) in a chemiluminescence imaging system (Amersham Imager 680). ImageJ software was used to determine the gray values of the bands. The antibodies used for the present study and the dilutions ratios used were as follows: PDPN (Catalog #YT6610, ImmunoWay, ImmunoWay Biotechnology Company, 1:2,000), CLEC2 (Catalog #YN2186, ImmunoWay, ImmunoWay Biotechnology Company, 1:1,000), PI3K (Catalog #ab191606, Abcam, 1:2,000), AKT (Catalog #ab179463, Abcam, 1:1,000), mTOR (Catalog #2983, Cell Signaling Technology, 1:1,000), and glyceraldehyde-3-phosphate dehydrogenase (GAPDH) (Catalog #10494-1-AP, Proteintech, Wuhan Sanying Biotechnology Co., Ltd., China, 1:8,000).

### Isolation of spleens, MLNs, and tumor cells

For anti-tumor immunity analysis, the spleen, MLNs, and tumors were removed, cut into small pieces, washed in PBS, mechanically triturated with a syringe, and filtered through a 200-mesh filter. Single-cell suspensions of spleen and tumor cells were extracted with a mouse spleen lymphocyte separation liquid kit (Catalog #P8860; Beijing Solarbio Science & Technology Co., China) and a mouse tumor infiltration tissue lymphocyte separation liquid kit (catalog # P9000; Beijing Solarbio Science & Technology Co., China), respectively. Finally, the cell suspension was passed through a 200-mesh filter before being centrifuged for 5 minutes at 1,500 rpm. The isolated cells were analyzed further.

### Flow cytometry analysis

After the cells were extracted from the spleen, the MLNs and tumors were counted, and a 100 µL cell suspension containing 1 × 10^6^ cells was prepared for flow cytometry staining. For surface staining, the cells were stained with 0.3 µL of antibody. For intracellular cytokine staining, the cells were permeabilized with the eBioscience Foxp3/Transcription Factor Staining Buffer Set (Catalog #00-5523-00; Thermo Fisher). Transcription factor staining buffer (200 µL) was added, after which the samples were incubated at 4°C for 25 minutes in the dark, followed by incubation with 1 µL of intracellular antibodies in a refrigerator at 4°C for 30 minutes in the dark. Surface staining was performed using the following antibodies: F488-conjugated anti-CD8 antibody, phycoerythrin (PE)-conjugated anti-PDPN antibody, fluorescein isothiocyanate (FITC)-conjugated anti-CD4 antibody and BV510-conjugated anti-CD25 antibody. Intracellular staining was performed using the following antibodies: BV421-conjugated anti-TNF-α antibody, PerCP-Cy 5.5-conjugated anti-IFN-γ antibody, PE-conjugated anti-granzyme-B antibody, PE-conjugated anti-perforin antibody, and PerCP-Cy 5.5-conjugated Foxp3 antibody. Samples were analyzed using a flow cytometer (BD FACSCelestaTM). Subsequent analysis was performed with FlowJo software. Flow cytometry data analysis incorporated unstained and single-stained controls to enable accurate gating and compensation.

### Quantitative detection of SCFAs

The levels of fecal SCFAs, including acetic acid, butyric acid, isovaleric acid, valeric acid, caproic acid, isobutyric acid, and propanoic acid, were quantified via gas chromatography–mass spectrometry (GC‒MS) analysis (Thermo Fisher Scientific, USA) performed by PANOMIX (Suzhou PANOMIX Biomedical Tech Co., Ltd., China). GC analysis was performed on a trace 1,310 gas chromatograph (Thermo Fisher Scientific, USA). Mass spectrometric detection of metabolites was performed on an ISQ 7000 (Thermo Fisher Scientific, USA) in electron impact ionization mode. This process included the preparation of standard solutions, sample preparation, and calculation.

### Statistical analysis

Statistical analysis was performed using IBM SPSS Statistics 25 software (https://spss.mairuan.com/). Comparisons between two groups were performed using a two-sided Student’s *t* test. Analysis of variance (ANOVA) was used to compare differences among multiple groups. The values are expressed as the means ± standard deviations for both *in vivo* and *in vitro* experiments. A *P*-value less than 0.05 was considered to indicate statistical significance. The number of asterisks indicates the level of significance: *P < 0.05, **P < 0.01, ***P < 0.001.

## Data Availability

All data generated or analyzed during this study are included in this published article and its supplemental material files. All relevant data and materials can be obtained from the first author and corresponding authors.

## SUPPLEMENTAL MATERIAL

Supplemental figures msystems.00148-25-s0001.pdf Fig. S1 to S4.

Supplemental material msystems.00148-25-s0002.pdf Images of the original western blots.

ASM does not own the copyrights to Supplemental Material that may be linked to, or accessed through, an article. The authors have granted ASM a non-exclusive, world-wide license to publish the Supplemental Material files. Please contact the corresponding author directly for reuse.
