# Supplementary material for: Gut-derived Faecalibaculum rodentium exerts anti-cancer effects on colorectal cancer by modulating PDPN-CLEC-2 signaling pathway
Source: mSystems. 2025 Jul 22;10(8):e00148-25. doi: 10.1128/msystems.00148-25 (PMC12363190; doi:10.1128/msystems.00148-25)
Supplement: Supplemental figures — Fig. S1 to S4. [file msystems.00148-25-s0001.pdf]

Supplementary Figure

Fig. S1

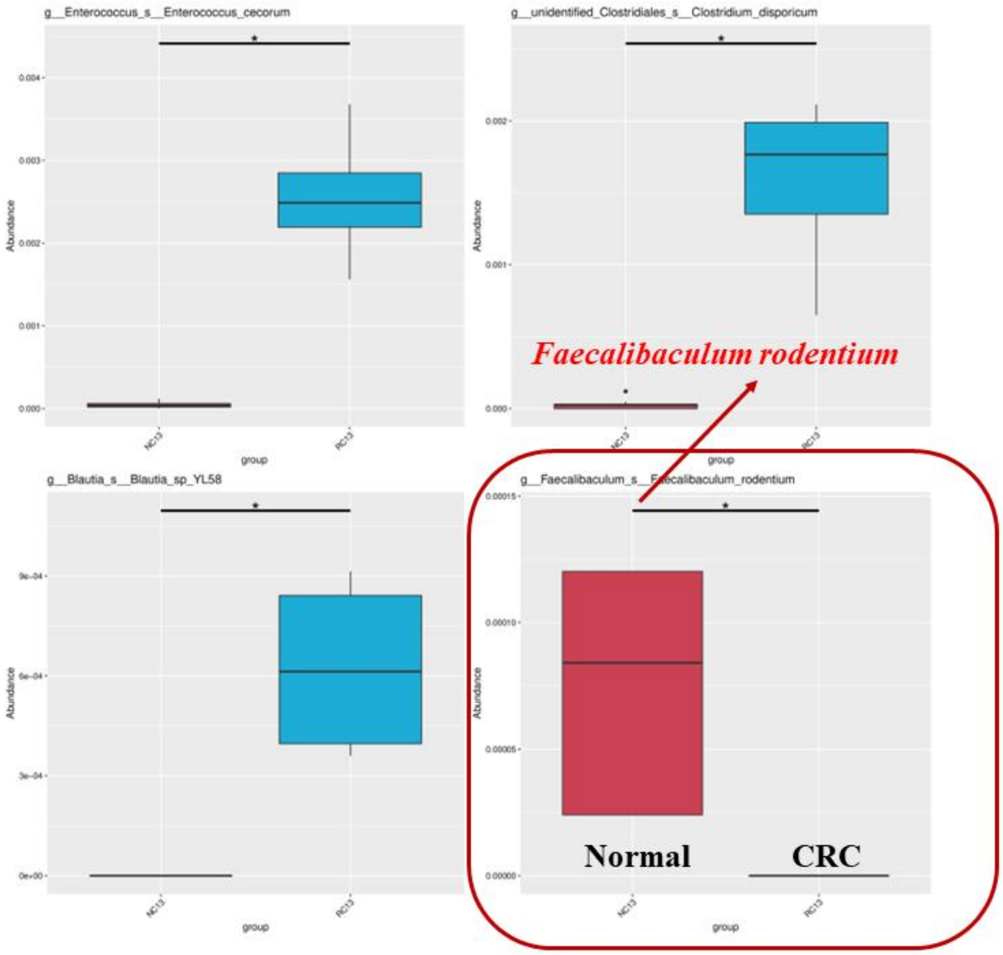

Figure S1. The abundance of *F. rodentium* decreased significantly in mice with CRC. A meta-analysis of 16S rRNA gene sequencing data from faecal bacteria in an AOM/DSS-induced CRC mouse model at the 13th week was performed. The abundances of *F. rodentium* in feces were compared using an unpaired Student's t-test. A two-tailed alpha level of 0.05 was used to determine statistical significance. P-values are presented as follows:ns, not significant; *p < 0.05; **p < 0.01; ***p < 0.001.

Fig. S2

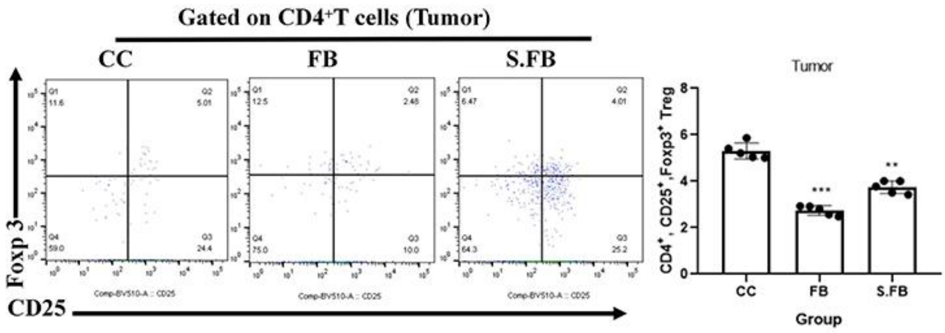

Figure S2. Effect of *F. rodentium* on Treg . Representative flow cytometry data for CD4+ CD25+ Foxp3+ Treg cell infiltration in the tumors. Plots were gated on CD4+ T cells. The frequencies of Treg cells were compared using one-way ANOVA . Data are presented as mean ± SD (n=5 mice per group). A two-tailed alpha level of 0.05 was used to determine statistical significance. P-values are presented as follows:ns, not significant; *$p < 0.05$; **$p < 0.01$; ***$p < 0.001$.

Fig. S3

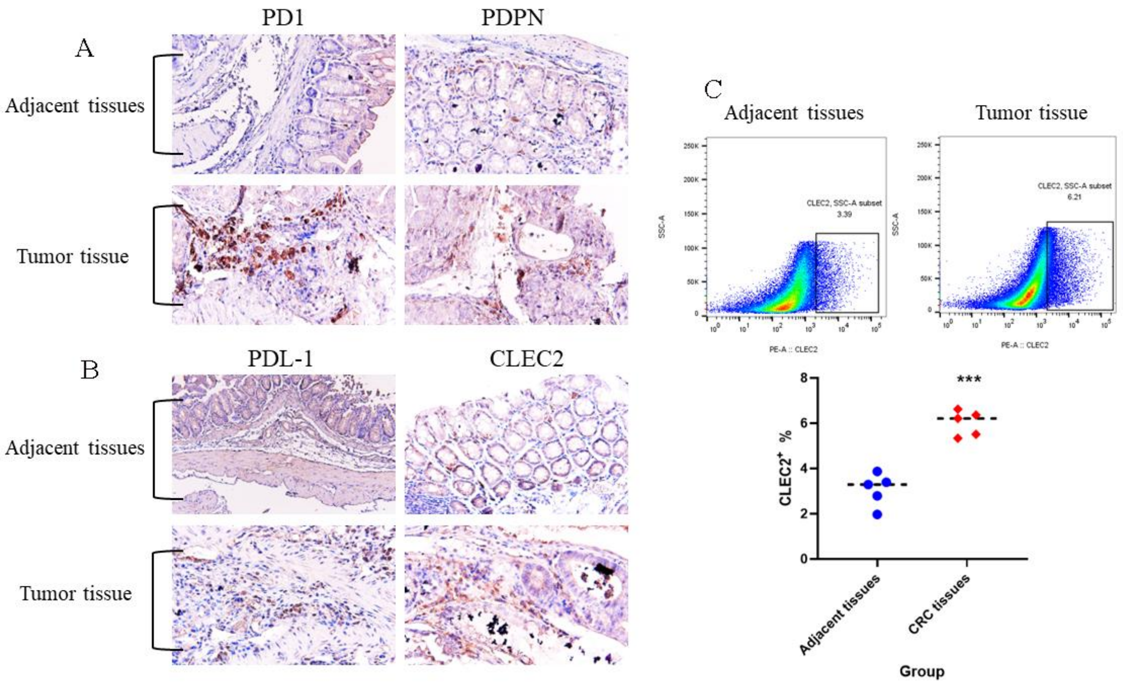

Figure S3. PDPN expression on lymphocytes and CLEC-2 expression on tumour cells were significantly increased in CRC mouse tumour tissues induced by AOM/DSS. (A) PDPN and PD1 immunohistochemical staining of tumour tissue at the 13th week was performed. Scale bar: 50 µm. **(B)** CLEC2 and PDL-1 immunohistochemical staining of tumour tissue at the 13th week was performed. Scale bar: 50 µm. **(C)** Representative flow cytometry data of CLEC2 + tumour cell infiltration in tumours at the 13th week were obtained. The frequencies of CLEC2 + cells in tumour tissues were compared using an unpaired Student's t-test. Data are presented as mean ± SD (n=5). A two-tailed alpha level of 0.05 was used to determine statistical significance. P-values are presented as follows:ns, not significant; *$p < 0.05$; **$p < 0.01$; ***$p < 0.001$.

Fig. S4

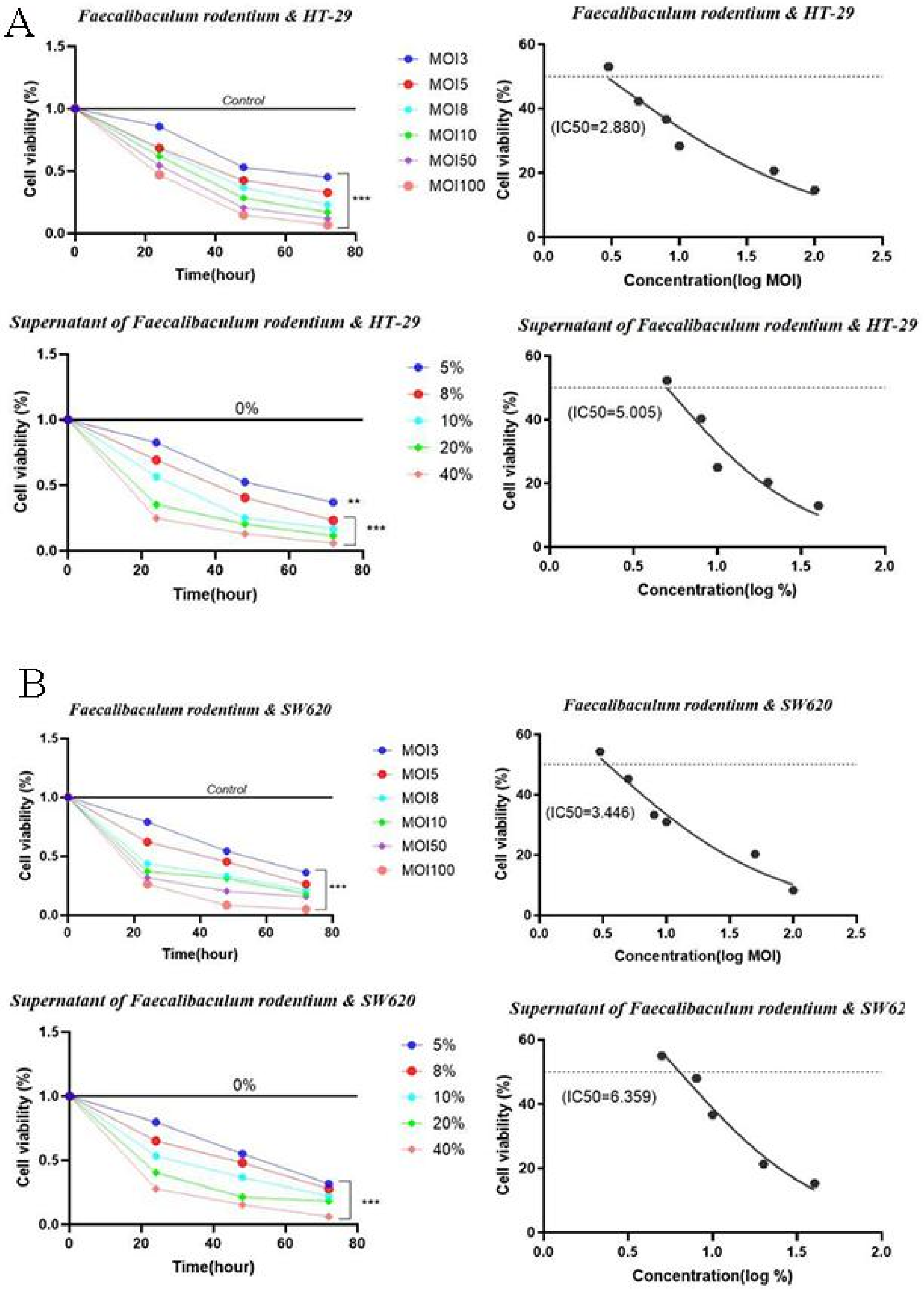

Figure S4. F. rodentium and its metabolites suppress colorectal cancer cell viability in a concentration-dependent manner. (A) Cell viability of HT-29 cells following treatment with *F. rodentium* or its metabolites at various concentrations for 24, 48, and 72 hours, as determined by the CCK-8 assay. (B) Cell viability of SW620

cells following treatment with *F. rodentium* or its metabolites at various concentrations for 24, 48, and 72 hours, as determined by the CCK-8 assay. Data were analyzed using repeated measures ANOVA to assess the effects of *F. rodentium* and its metabolites concentrations on cell viability. A two-tailed alpha level of 0.05 was used to determine statistical significance. P-values are presented as follows:ns, not significant; *$p < 0.05$; **$p < 0.01$; ***$p < 0.001$.
