# Supplementary material for: Gut-derived Faecalibaculum rodentium exerts anti-cancer effects on colorectal cancer by modulating PDPN-CLEC-2 signaling pathway
Source: mSystems. 2025 Jul 22;10(8):e00148-25. doi: 10.1128/msystems.00148-25 (PMC12363190; doi:10.1128/msystems.00148-25)

Images of the original western blots

1.The following image represents western blot analysis shown in **Fig 2F** (**PDPN**)

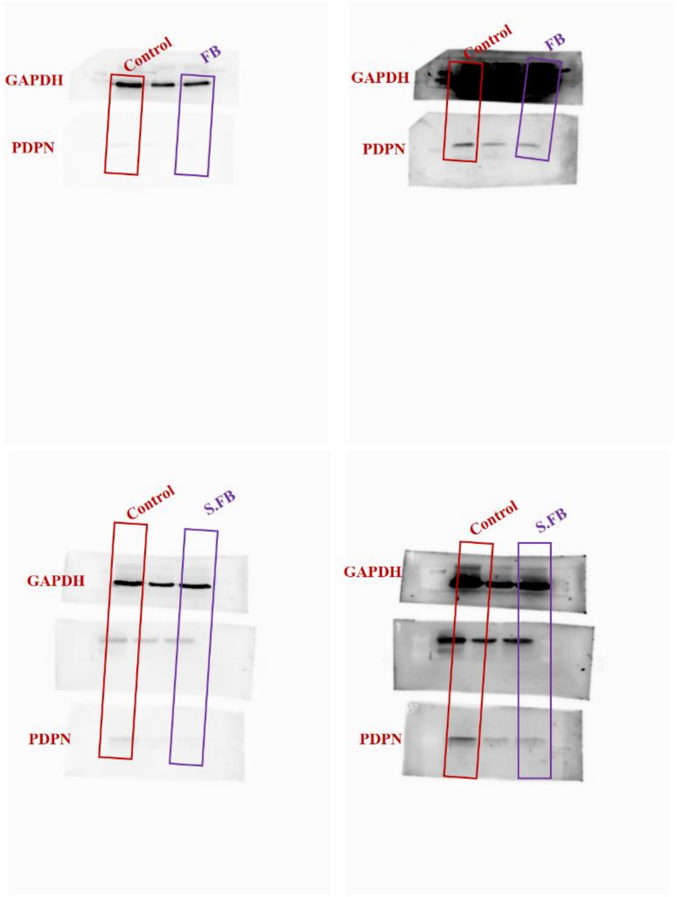

2.1The following image represents western blot analysis shown in Fig **4A** (**CLEC2**)

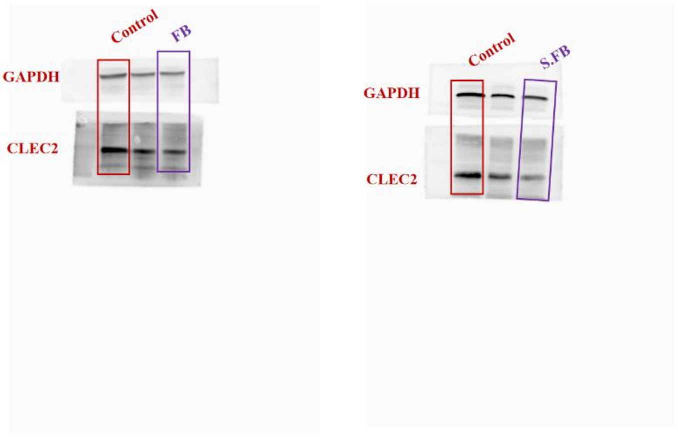

2.2 The following image represents western blot analysis shown in Fig **4A** (**PI3K**)

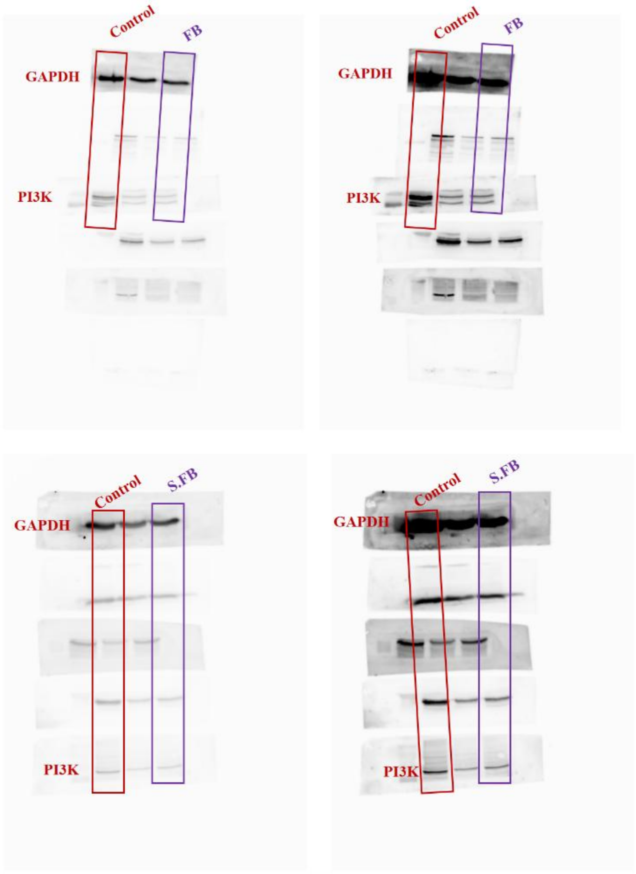

2.3 The following image represents western blot analysis shown in Fig **4A** (**AKT**)

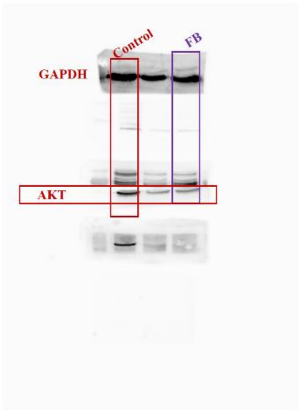

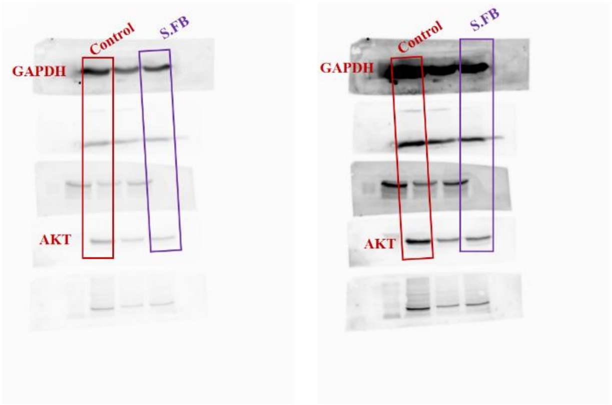

2.4 The following image represents western blot analysis shown in Fig **4A** (mTOR)

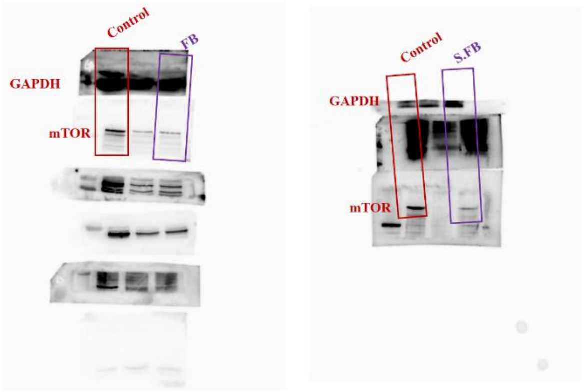

3.1 The following image represents western blot analysis shown in Fig **4B** (CLEC2)

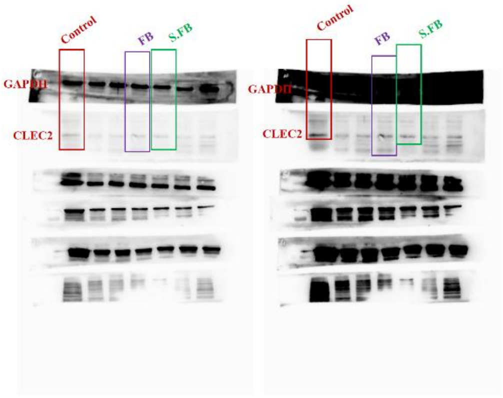

3.2 The following image represents western blot analysis shown in Fig **4B** (PI3K)

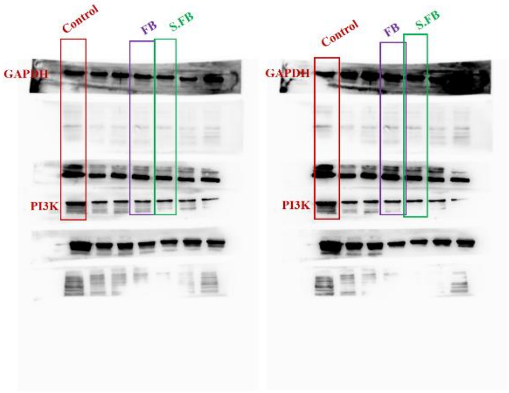

3.3 The following image represents western blot analysis shown in Fig **4B** (AKT)

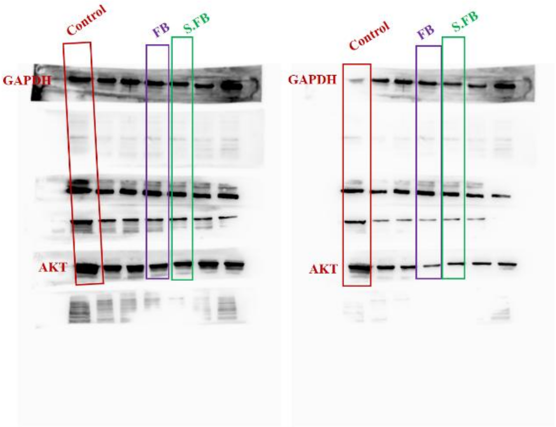

3.4 The following image represents western blot analysis shown in Fig **4B** (mTOR)

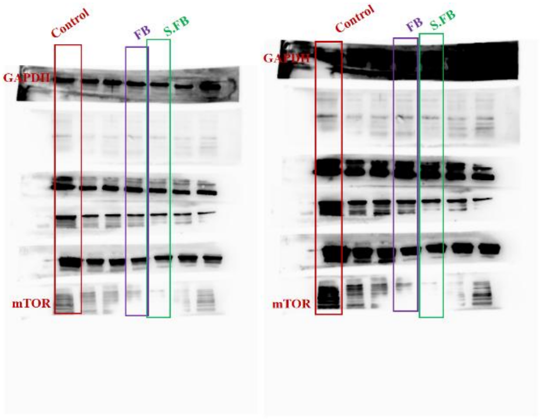

Supplement: Supplemental material — Images of the original western blots. [file msystems.00148-25-s0002.pdf]
